# A brief historical overview of the anatomy of fascia in medieval Persian medicine

**DOI:** 10.18502/jmehm.v13i7.4073

**Published:** 2020-08-25

**Authors:** Mohsen Bahrami, Saeed Shokri, Reza Mastery Farahani, Majid Dadmehr

**Affiliations:** 1 *Researcher, Tehran, Iran.*; 2 *Associate Professor, Department of Anatomical Sciences, School of Medicine, Zanjan University of Medical Sciences, Zanjan, Iran.*; 3 *Associate Professor, Department of Biology and Anatomical Sciences, School of Medicine, Shahid Beheshti University of Medical Sciences, Tehran, Iran.*; 4 *Assistant Professor, School of Persian Medicine, Iran University of Medical Sciences, Tehran, Iran; Research Institute for Islamic and Complementary Medicine, Iran University of Medical Sciences, Tehran, Iran.*

 The study of fascia has a long history and has been considered by physicians of ancient civilizations. A review of historical medical manuscripts demonstrates a rich history of anatomy in ancient and medieval times, for instance in medieval Persia (1-3). As one of the firstborn schools of medicine, Persian medicine (PM) has a long history and was the principal medical source during the Islamic golden age (from the 8^th^ to the 13^th^ century AD) in the medieval period (4,5). Medieval Persian practitioners were aware of other available medical traditions like those of ancient Greece, Egypt, India and China, and mostly applied the theories of Hippocrates and Galen as well. Moreover, they added numerous valuable scientific theories to medieval medicine according to their observations and experiments (5). The textbooks of some PM scholars, for instance Avicenna’s *Canon of Medicine*, were used by scientists and physicians in Europe until the seventeenth century AD (4). There were a number of influential Persian physicians who had years of experience in human dissection such as Rhazes (865 - 925 AD), Haly Abbas (930 - 994 AD), Avicenna (980 - 1037 AD) and Jorjani (1042 - 1137 AD). They had a significant role in the development of anatomy applied in patient care (4,5). PM scientists were familiar with the anatomy and interrelationships of fascia, and widely used this anatomical term. According to PM manuscripts, fascia is classified as a seminal organ (relating to semen or *Manavi*), which has been included in the category of nervous tissue with different functions. Fascia is associated with tissue nourishment, protection from the invasion of harmful agents and transmission of innate heat, spirits and energies among organs; moreover, it is believed that fascia makes a significant contribution to the underlying mechanisms and also the treatment methods of some diseases (1 - 3).

According to PM textbooks, fascia is classified as a simple organ originating from parental semen. Anatomically, body organs are categorized into two main groups: simple and compound. Organs that are created from a single material are called simple (e.g. bones, fascia and vessels), while compound organs are made up of a combination of simple organs (e.g. hand and eye) (2,3). Simple organs are classified into two main subgroups:

1- Organs that are formed from blood, that is, sanguine organs, such as *lahm* or flesh (flesh, muscles and glandular tissue) and *shahm* or adipose tissue (fat tissue).

2- Organs that are formed from semen, that is, seminal organs, such as bones (bones, cartilage and joints), vessels (arteries, veins and lymphatic vessels) and nerves (nerves, ligaments, tendons and fascia); therefore, according to the perspective of PM scientists, fascia is classified as the main component of nervous tissue (2, 3).

In the *Canon of Medicine,* Avicenna has defined fascia as nerve-like, transparent, thin, rigid and woven wide fibers that surround body organs and cover their external surface (3). 

Haly Abbas elucidated that fascia is a thin and rigid object that encloses the organs, and there is no tissue thinner than it. He believed that after the bone, fascia is the most rigid tissue in the body, covering and maintaining the related organs and protecting them from the invasion of harmful agents; therefore, fascia is rigid so it will not be easily affected by harmful agents, and it is thin so it will not occupy a large space in the human body (2).

PM scientists have used several terms such as *Bitanah* (lining), *Ghilaf* (sheath), *Ma’aliq* (ligament) and *Ghisha* (membrane or fascia) to introduce superficial, deep and visceral fasciae (1 - 3). The natures of these fasciae are not different from each other histologically; however, superficial fascia is general, but deep and visceral fascia is specialized. It has also been mentioned that the components and composition of the fasciae and their functions are different in various tissues (2,3). Haly Abbas believed that body parts such as muscles have a single layer membrane, while internal organs (the viscera) are covered in a double layer of fascia: the parietal layer envelopes the walls and the visceral layer covers those organs (2).

According to PM textbooks, fascia can be divided into four general groups based on their locations in the human body:

*1-   Fascia of the brain and spinal cord*

Two fasciae called *Omm ol-jafiyya* (dura mater) and *Omm or-raqiqa* (pia mater) surround the brain entirely. Dura mater is joined to the fissures of skull bones and pia mater penetrates and is distributed all over the brain tissue. Pia mater along with the fascia around the brain vessels create a structure like *Mashimah* (chorion), while a third fascia spreads from the skull bones to the lower end of the vertebral column (2, 3). 

*2-   Fascia of the chest*

Rhazes and Avicenna pointed out that a thin spidery fascia located on the inner surface of the ribs (chest wall) covers the ribs and the internal organs of the chest (endothoracic fascia). Two fasciae originate from this fascia and split the chest into two parts, joining the sternum from one side and covering the heart, lungs, blood vessels and nerves, and on the other side, they join the vertebrae on top of the esophagus (1, 3).

*3-   Fascia of the abdomen*

According to Avicenna, the abdomen has two fasciae: *Maraqq*, or the external layer or parietal peritoneum, and *Baritun*, or the visceral peritoneum. *Maraqq* is the fascia surrounding the digestive tract and is located under the skin and abdominal muscles. *Baritun* is a thin, fragile and spidery fascia lining the abdominopelvic organs (stomach, liver, spleen, kidneys, bladder, uterus, testicles, omentum, vessels, nerves and so on) (3).

4-   *Fascia surrounding the fetus*

Haly Abbas explained that this fascia consists of three layers, each of which has a specific function as follows (2):

     ˗    The outermost layer surrounding the fetus is *Mashimah* (chorion), which is responsible for nourishment of the fetus

     ˗    The second one called *Seqa* is located inside the chorion

     ˗    The third layer named *Sela* is located inside the *Seqa*

PM scientists like Haly Abbas and Avicenna have mentioned several functions for fascia, some of which include enclosure and coverage of body organs, communication and mediation among organs, creating a mechanical barrier, splitting up an organ and preserving innate heat and spirits (2, 3).

Furthermore, Haly Abbas pointed out other functions for the pia mater including:

     ˗    Connecting brain vessels so that they are fixed and are not loose 

     ˗    Covering the brain and protecting it from damage

     ˗    Feeding the cerebral veins and conveying the innate heat to the arteries (2)

Based on the information provided by Avicenna, the peritoneum has some other functions:

     ˗    Enclosing the viscera under the diaphragm

     ˗    Preserving the abdominal muscles from falling over the viscera and bladder

     ˗    Contributing to peristalsis (with the help of abdominal muscles)

     ˗    Joining together all the viscera under the diaphragm (each of them has a separate fascia with its own properties) (3)

Medieval Persian scholars were aware of the widespread presence of fascia in all body tissues. Fascia plays an important role in underlying mechanisms and corresponding therapies of a number of diseases in different organs (1-3). According to the *Canon of Medicine*, there is a relationship between brain and abdomen through fasciae and nerve sheaths (3). Therefore, in patients who experience certain gastrointestinal manifestations, a higher prevalence of brain diseases such as epilepsy, headache and melancholia may occur (3). Moreover, topical application of medicinal oils and abdominal massage are recommended for treatment of flatulence, lienteric diarrhea, constipation and bladder prolapse (3). In some gastric diseases, use of abdominal massage and applying oils through the anterior abdominal wall have been recommended (2, 3). These techniques have been mostly recommended in sick and elderly people who experience gastric disorders and constipation due to the weakness of abdominal wall muscles, fasciae and ligaments, as it is believed that these fasciae contribute to peristalsis with the help of abdominal muscles (2,3). Avicenna has also mentioned a variety of headaches such as those related to the neck, kidneys, liver and spleen disorders, adding that the fascia network contributes to their pathophysiology (3).

In some diseases, massage and manual therapy are used on the vertebral column to erode some of the confined cerebral wastes through the fascia surrounding the spinal cord (3). Avicenna has dissected the covering layers of the parietal and visceral peritoneum and has explained the connection between peritoneum and testicular covering sheath through the inguinal hole. In addition, he has discussed the types of hernias in this area and their respective treatment methods (3).

According to the principles of PM, the clinical effects of some treatment methods such as massage therapy, cupping therapy and anointing with oils are justified through the concept of fascia. 

In conclusion, PM scientists were conscious of the widespread presence, as well as the supportive and protective role of fascia throughout the entire human body (1-3). According to PM manuscripts, fascia is a seminal organ and in the category of nervous tissue with different functions that contributes to tissue nourishment and transmission of innate heat, spirits and energies among organs (2, 3).

Existing embryological evidence shows that the fascia network originates from the mesoderm, although some authors believe that this connective tissue is also found in the neural crests (ectoderm), especially in the cranial and cervical area (6).

PM scientists believed that fascia has a communicating and mediating role among body organs and plays a significant role in the treatment of a number of diseases. They used dry cupping, massaging and anointing oil on the abdominal wall as a treatment method for certain diseases (2,3). 

There is a bidirectional interaction among fasciae and every muscle, organ, blood vessel and nerve (7). Some evidence proposes that the fascia network has a key role in manual therapy and acupuncture (7,8). It seems that fascia can offer a pathophysiological basis for clarifying the clinical effects of these treatments, since it can be considered as a connection between the surface and the viscera on the depth of the body. This can provide the possibility of treating visceral diseases through the body surface. The fascia reacts to stimulation, and therapeutic efficacy is therefore clearly linked to accurate fascial localizations (7). 

## Notes:

**Citation to this article:**

Bahrami M, Shokri S, Mastery Farahani R, Dadmehr M. A brief historical overview of the anatomy of fascia in medieval Persian medicine. J Med Ethics Hist Med. 2020; 13: 7.
